# Response to peginterferon plus ribavirin and subsequent retreatment with telaprevir-based triple therapy in a patient with chronic lymphocytic leukaemia and chronic HCV genotype 1b infection

**DOI:** 10.1186/1750-9378-9-10

**Published:** 2014-03-20

**Authors:** Stefan Christensen, Anton Gillessen

**Affiliations:** 1 Infektiologische Praxisgemeinschaft, Salzstrasse 58, D-48143 Münster, Germany; 2 Herz-Jesu-Krankenhaus Klinik für Innere Medizin, Salzstrasse 58, D-48143 Münster, Germany

**Keywords:** Chronic hepatitis C, Chronic lymphocytic leukaemia (CLL), Peginterferon, Telaprevir, Late relapse, Retreatment, SVR

## Abstract

**Background:**

Case-controlled studies have clearly demonstrated a link between chronic hepatitis C infection (CHC) and B cell non-Hodgkin lymphoma (NHL). To our knowledge, this is the first case report of outcome in a patient with CLL and chronic HCV infection treated with PEG-IFN/RBV and subsequent retreated with triple therapy.

**Findings:**

We report the case of a 54-year old, caucasian woman with a history of elevated liver enzymes diagnosed with chronic lymphocytic leukaemia (CLL) detected during investigation for hepatitis C (HCV) infection. The patient showed a haematological response following initially successful anti-HCV therapy with peginterferon plus ribavirin (PEG-IFN/RBV), with normalization of leukocyte and lymphocyte counts. She subsequently showed a late virological relapse at week 24, and was successfully retreated with telaprevir-based triple therapy. Despite an increase in leucocyte and lymphocyte count compared to baseline following triple therapy, to date there is no evidence of progression of CLL and the patient remains asymptomatic.

**Conclusion:**

Patients with CLL may experience haematological response following successful anti-HCV therapy using IFN-based regimens. Re-treatment with triple therapy including telaprevir following late virological relapse was successful, was not associated with any unexpected safety issues, and did not adversely affect CLL status.

## Background

Case-controlled studies have clearly demonstrated a link between chronic hepatitis C infection (CHC) and B cell non-Hodgkin lymphoma (NHL) [1]. This evidence has been supported by an increasing number of reports showing a relationship between haematological response in patients with a range of NHLs and sustained virological response (SVR) following interferon (IFN)-based anti-hepatitis C virus (HCV) therapy [2-4]. Although rare, virological relapse following a sustained response post-treatment with IFN-based therapy does occur [5]. Recent treatment guidelines recommend the use of triple therapy with peginterferon plus ribavirin (PEG-IFN/RBV) and either telaprevir or boceprevir (approved for use in Germany in 2011) in both treatment-naive genotype-1 infected patients and for those who have failed previous PEG-IFN/RBV therapy [6,7]. The effect of recombinant interferons on B-lymphocytic lymphoma is well known for years [8] and recent studies show that aberrant interferon-signaling is associated with aggressive CLL [9]. To date, there are no reports of the use of triple therapy for chronic HCV infection in patients with NHL.

We present a report of a case of chronic lymphocytic leukaemia (CLL) in the context of chronic HCV infection treated with PEG-IFN/RBV, and subsequently retreated with triple therapy following late virological relapse.

## Case report

A 54 year old, caucasian woman with a 12 year history of elevated liver transaminases was referred for investigation in March 2010. The patient complained of recurrent vertigo but was otherwise asymptomatic. Her medical history included arterial hypertension diagnosed 5 years previously and managed using bisoprolol, ramipril and lercandipin. She was a non-smoker, did not drink alcohol, and had no known allergies. Her surgical history comprised appendectomy and tonsillectomy in childhood, and surgery for a prolapsed intervertebral disc and breast reduction surgery, both approximately 15 years previously.

Initial testing revealed that the patient was anti-HCV positive. Further testing showed genotype 1b CHC infection, interleukin (IL) 28B C/T and inosine triphosphatase (ITPA) CC/AA genotypes, and a low viral load (HCV RNA 53049 IU/mL; HCV Abbott RealTime PCR version 4.0, lower limit of quantification 12 IU/mL; Abbott GmbH & Co., Wiesbaden, Germany). Gamma-glutamyl transpeptidase (gamma-GT) levels were found to be elevated (62 U/L, normal >39 U/L) as were her alanine aminotransferase (ALT) and aspartate aminotransferase (AST) (88 U/l and 76 U/L, respectively; normal for both <35 U/L) levels. Other liver function indicators including albumin, international normalised ratio (INR) and serum cholinesterase activity (CHE) were normal. Transient elastography (FibroScan®) showed liver stiffness of 11.6 KPa, indicative of fibrosis (F3), although this may have been overestimated due to the presence of non-alcoholic steatohepatitis on ultrasound.

In addition to the diagnosis of CHC, the patient was incidentally found to have leucocytosis (15900/μl) and lymphocytosis (8200/μl). Bone marrow biopsy confirmed a diagnosis of CD20- and CD78-positive CLL with 5-10% infiltration of the bone marrow, Binet stage A. The patient was asymptomatic and no treatment for CLL was indicated at this timepoint.

Given the patient’s age and fibrosis grade, her willingness to undergo therapy and uncertainty associated with the possible progression of her CLL, it was decided to initiate antiviral treatment in August 2010 with peginterferon (PEG-IFN) alfa-2a (PEGASYS®; Roche Pharma AG, Grenzach-Wyhlen, Germany) 180 μg/weekly plus ribavirin (RBV; COPEGUS®; Roche Pharma AG, Grenzach-Wyhlen, Germany) 1000 mg/day. At this time, neither telaprevir nor boceprevir were approved for HCV infection in Germany.

On-treatment, her viral load declined to 818 IU/mL by week 4 and was undetectable by week 12, consistent with a complete early virological response (cEVR). Her transaminases remained elevated (ALT 64 U/L; AST 65 U/L). Leucocytes decreased to 4600/μl with lymphocyte count 2346/μL. On treatment, the patient experienced anaemia (haemoglobin 10 g/dL) which was managed without RBV dose reduction, and hypothyroidism with elevated thyroid-stimulating hormone (TSH) levels in the absence of detectable antibodies, which was treated with L-Thyroxine 50 μg/d.

At end of treatment (Week 48) and at Week 12 post-treatment, viral levels remained below limit of detection (SVR12). At week 12 liver transaminases were slightly elevated (ALT 37 U/L; AST 43 U/L), and both leucocytes (9600/μl) and lymphocytes (3216/μL) were within the normal range. A control bone marrow biopsy performed one month later showed immunhistochemical evidence of nodular lymphocyte infiltrates as residue of a small-cell lymphocytic B-cell lymphoma with infiltration density of 25% without evidence of transformation to large cell lymphoma.

In January 2012, 24 weeks post-treatment, there was an increase in HCV RNA (102817 IU/mL) with elevated ALT (55 U/L) and AST (52 U/). As there was no indication of any risk for re-infection, the patient was diagnosed with late relapse. At this time, leucocytes (7800/μl) and lymphocytes (3260/μL) remained within the normal range.

As the patient was still willing to undergo therapy, re-treatment with a triple therapy regimen was initiated in March 2012. At this time her liver stiffness by FibroScan® was 9.2 KPa (F2/F3). The patient initially received PEG-IFN alfa-2a 180 μg/week plus RBV 1000 mg/d plus telaprevir (Incivo®; Janssen Cilag International NV, Beerse, Belgium) 750 mg every 8 hours. After 12 weeks of triple therapy, treatment was continued with PEG-IFN alfa 2a plus RBV alone for a further 12 weeks (total treatment duration 24 weeks). On treatment, her viral load declined to 12 IU/mL after 14 days and was below limit of detection by week 4. In week 2, RBV dose was reduced to 800 mg/day following a decline in haemoglobin by 2.6 g/dL to 10.3 g/dL; PEG-IFN alfa 2a dose remained unchanged throughout treatment. There was no requirement for growth factors or for erythrocyte concentrates. Side effects of treatment included nausea, recurrent vomiting, fatigue, and mood changes, including depressive symptoms which were managed using citalopram. The patient experienced a localized skin reaction with itching on her back early in triple therapy which was easily managed with corticosteroids, and was no longer a problem after week 12.

Post-treatment, HCV RNA remained undetectable at both Week 12 and Week 24 (SVR12 and SVR24), and ALT and AST levels were within the normal range. However, the patient developed leucocytosis (13100/μL) again with a lymphocyte count of 6485/μL. There was no evidence for progression of CLL and the patient remained clinically asymptomatic.

At her most recent presentation (March 2013), the patient’s HCV RNA levels remain undetectable, and her liver enzymes are within normal ranges. Although her leucocyte and lymphoctye counts remain elevated, she is still asymptomatic, with has no indication for CLL therapy and continues under observation by her haemato-oncologist.

## Discussion

To our knowledge, this is first report describing the outcome of a patient with CLL and CHC with a late virological relapse after dual combination treatment with PEG-IFN plus ribavirin, and retreatment with triple therapy including telaprevir leading to a sustained virological response. In addition, this case report emphasizes the importance of post-treatment monitoring of response to therapy with PEG-IFN plus ribavirin, and describes successful retreatment with triple therapy following a late relapse.

There is growing evidence from a number of large case-controlled studies of an association between HCV infection and the development of non-Hodgkin lymphoma (NHL) [1]. This link is particularly strong for B-cell NHL including diffuse large B lymphoma, marginal zone lymphoma and small lymphocytic lymphoma/CLL. HCV prevalence has been reported to be around 15% in patients with B-cell NHL, compared with 1.5% in the general population, although there is marked geographic variation [10,11]. In a meta-analysis of case-controlled studies, HCV infection was found to be strongly associated with risk of NHL, with an odds ratio of 5.04 (95% confidence interval (CI), 4.09-7.96, P < 0.001) [12]. Additional evidence for an association between HCV infection and NHL comes from a number of studies reporting the effect of antiviral therapy on lymphoma. Complete or partial haematological remission has been reported following IFN-based therapy for B cell NHLs, particularly in marginal zone lymphomas [2-4]. In a study of patients with a range indolent B-cell NHLs, more patients achieved a response (complete or partial) following treatment with PEG-IFN/RBV (80%) compared with convention IFN/RBV [4]. Haematological response has been shown to be linked to achievement of an SVR, and to be maintained as long as HCV infection does not reoccur [3,4]. No response to IFN is reported in a systematic review of Gisbert et al. in HCV-negative patients with lymphoproliferative diseases, indicating that the response in HCV-positive patients is clearly not merely due to antiproliferative effects of IFN [3].

To our knowledge, there is currently only a single report in the literature of CLL with hepatic involvement and HCV infection which responded to PEG-IFN therapy [13]. In keeping with reports in other patients with B cell NHL, the disappearance of HCV RNA during initial PEG-IFN/RBV treatment in our patient was associated with a haematological response in the patient’s CLL, which was maintained post-treatment. Although Interferon is not part of the standard therapy anymore, our observations are in line with several older studies which showed some treatment effects of interferon in CLL resulting in a haematological response [14-16]. This haematological response persisted at post-treatment week 24, where virological relapse was detected. Retreatment with triple therapy with telaprevir plus PEG-IFN/RBV following virological relapse did not result in any safety issues with respect to the patient’s CLL. Although the patient’s leucocytes and lymphocytes were elevated following triple therapy despite a sustained virological response, there remains no indication of progression of her CLL and she remains asymptomatic without treatment. It is unclear why the ongoing sustained virological response in our patient following triple therapy has not been associated with a similar haematological response as was seen following PEG-IFN/RBV alone.

The late virological relapse seen after PEG-IFN/RBV therapy emphasizes the importance of post-treatment monitoring. Late relapse is rare (<1%) following SVR at 24 weeks post-treatment (SVR24) and achievement SVR24 is generally regarded as a ‘cure’ following IFN-based therapy, associated with improvements in liver histology, liver-related morbidity and mortality [17]. There has been support for using 12 week post-treatment response to indicate SVR, and the Federal Drug Agency (FDA) now recommends use of SVR12 as the primary end point for HCV clinical trials [5,18,19]. However, relapse between post-treatment week 12 and 24 was seen in around 2% of patients overall and 5% of genotype 1-infected patients [5]. While SVR12 may be relevant in registrational clinical trials, in the clinic continued monitoring at week 24 post-treatment is still relevant to identify those rare patients, such as ours, with later relapse, to allow for early retreatment where applicable.

## Conclusions

As in other NHLs, patients with CLL may experience haematological response following successful anti-HCV therapy using IFN-based regimens. Re-treatment with triple therapy including telaprevir following late virological relapse was successful, was not associated with any unexpected safety issues, and did not adversely affect CLL status.

### Consent

Written informed consent was obtained from the patient for publication of this case report. A copy of the written consent is available for review by the Editor-in-Chief of this journal.

## Competing interests

SC has received speaker’s fees from Roche Pharma AG and Janssen-Cilag GmbH, and has participated in Advisory Boards for Roche Pharma AG and Janssen-Cilag GmbH. AG has no competing interests to declare.

## Authors’ contributions

Both authors participated in the acquisition of data, interpreted the data and participated in the drafting and revision of the manuscript. Both authors read and gave final approval for the version submitted for publication.
